# Editorial

**Published:** 1865-04

**Authors:** 


### Editorial.

VACCINATION REPORT.—The report on Vaccination, &c., in the original department of this number of the EXAMINER, was prepared by the Committee, and a printed copy sent simultaneously to all the medical periodicals of the country, with a request to give its facts as wide a circulation as possible. We not only give it a prominent place in the EXAMINER, but we hope it will be read, and its important suggestions acted upon by all our readers.

SUMMER MEDICAL INSTRUCTION.—The Summer Reading and Clinical Course of Instruction in the Chicago Medical College, is progressing regularly, with an interesting class of students in attendance.

OBITUARY.—DAVID RUTTER, M.D., of this city, died on the evening of the 16th of April, 1865, aged 65 years. He was attacked suddenly with apoplexy, about 9 o'clock P.M., and died in three or four hours. We copy the following account from one of the daily papers of this city:—

One of the oldest and most esteemed citizens of Chicago, Dr. David Rutter, was yesterday (April 20th) borne to the silent grave. In the death of this venerable and much respected gentleman, Chicago has lost one of its most worthy citizens, the medical profession one of its leading ornaments, and the bereaved family a kind husband and indulgent parent.

Dr. David Rutter was probably the oldest medical practitioner in the city, and no man stood more deservedly high in the estimation of the members of his profession. He was a native of Philadelphia, and lived in that city until his removal to this city, about sixteen years ago. In Philadelphia, he was intimately associated with the most prominent and eminent members of the medical profession, and no one was more beloved and admired. His well-earned reputation in the East at once brought him into an extensive practice upon his removal to Chicago; and his kindness, skill, and ability, quickly won for him here hosts of devoted friends.

A meeting of the members of the medical profession was held yesterday afternoon, in room No. 5, court-house, for the purpose of taking action relative to the decease of Dr. Rutter, and adopting resolutions expressive of their loss and sorrow. A large number of gentlemen were present. Dr. N. S. Davis was called to the chair, Dr. Brock McVickar was chosen secretary.

Upon taking the chair, Dr. Davis stated the object of the meeting, and, in a few well-chosen remarks, paid a high tribute to the name and memory of the deceased. Upon this sad occasion, he said, of uniting to take suitable action in relation to the death of one of their number, he felt constrained by a sense of duty, as well as by a feeling of pleasure, to give testimony to the worth and purity of the deceased. Little, however, need to be said relative to his talent, virtue, and ability, which were so well known to his brothers of the profession. Dr. Rutter was one of the oldest, most esteemed, and best beloved among them, with a mind acute, active, and enlarged by liberal study, a heart kind and sympathetic, and a disposition genial and forgiving. He had been always studiously observant of the rules and etiquette of the profession, and universally courteous to all.

In his lectures to the students at college, Dr. Rutter had never failed to inculcate the most exalted lessons of morality, while giving invaluable professional instruction. Dr. Davis concluded his remarks by expressing his deep sympathy for the grief-stricken family of the deceased.

The following gentlemen were then appointed a committee to draft resolutions expressive of the sense of the meeting, upon the sad occasion:—Dr. C. G. Smith, Dr. B. McVickar, and Dr. J. W. Freer.

After a short absence, the committee reported the following resolutions, which were unanimously adopted:—

*Resolved*, That as members of the medical profession of the City of Chicago, we desire to express our sense of the bereavement which we have suffered, socially and professionally, in the decease of our esteemed friend and colleague, Dr. David Rutter. That in his death we mourn the loss of a counsellor, wise from his ripe experience and long devotion to the duties of his profession; a friend whose precepts and whose principles we have always revered and admired, and whom we shall always cherish deeply in our memories as the wise physician, the true and steadfast friend, and noble Christian gentleman.

*Resolved*, That the shock which he received, and which caused his sudden death, when he learned the fate of our lamented President, is a testimony to his loyalty and love of country which words are powerless to express.

*Resolved*, That we sympathize with the friends and family of our deceased friend in their irreparable loss. He was to them a devoted husband and father, and around him clustered all the affections of a young and growing family, whose loss cannot be made up to them, and whose grief time only can assuage.

*Resolved*, That a copy of the foregoing resolutions be sent to the family of the deceased.

The meeting then adjourned.

---

Epidemic of Plague in St. Petersburg, Russia.—As the rise and progress of all severe epidemics are subjects of interest to intelligent medical men, we make no apology for copying the following from two secular London papers. For, though the statements of such papers are seldom correct or reliable in regard to medical matters, and especially in regard to the prevalence of diseases, yet they are sufficient to indicate the fact that a severe epidemic disease is prevailing in the capital and other parts of Russia, and Northern Europe. It is undoubtedly, as suggested by Dr. Murchison, of London, a form or modification of typhus. We think this latter disease has been increasing in most of the great commercial cities of the world for the last three years. The newspaper accounts are as follows:—

"An epidemic, resembling in its fatality the Asiatic cholera, has for some months devastated the interior of Russia. Apparently taking its origin in Siberia, it has gradually swept down southward, spreading more widely on either side as it advances. As yet it has completely baffled the skill of the Russian physicians, and of those professors of medicine who have proceeded from Germany to study its symptoms. In many respects this epidemic resembles the celebrated plague of Athens, which decimated Attica in the second and third years of the Peloponnessian war. Like it, the epidemic belongs to the class of eruptive typhoid disorders. The person seized immediately despairs of recovery; he loses memory and hope together. Like it, too, the Siberian fever is accompanied generally by a hoarse cough and violent retching, and the victim seldom survives beyond the ninth day. There is some difficulty in obtaining a reliable account of the disease, for the Russian officials, never very communicative, have endeavored to conceal the existence of the disease. But it has touched one or two towns in Austria and Prussia, and rages at St. Petersburg. The deaths in the latter city are acknowledged to amount to eighty or one hundred per day, but it is suspected they are five times as numerous. The disease is said to have assumed a mitigated form in Germany, but very great alarm prevails throughout the continent. Men hoped that with the Asiatic cholera the last great scourge of the human race had passed away, but they suddenly find themselves confronting a pestilence which advances as rapidly as a prairie conflagration, floating on the rivers, and borne on the air. Apprehension, too, as in the case of the Asiatic cholera, predisposes to the disease.

A plague of this description, raging in St. Petersburg, cannot be long absent from other European capitals. It marches steadily and surely. Already its route is traced by death and mourning, and its future track has been pointed out. In such a case quarantine regulations are nearly useless. No plague was ever yet kept away from our shores by delaying a ship from an infected port at a distance from the harbor. The fever may be conveyed in a letter, a bale of goods, a waif, or straw from the ship wafted to the shores. It may be taken up by the wind passing over the deck, and be borne mysteriously, despite of all precautions, to the crowded town. Physicians may dispute whether it is infectious or contagious. Such discussions may be interesting to the professor, but the practical truth is, that whether a plague be conveyed by the air or by contact, there is no means of staying its progress to any land to which it may be the will of Providence to visit with such a scourge." —*Liverpool Post.*

"Terrible Extent of the Ravages of the Disease.—The total number of cases has reached ten thousand, of deaths two thousand, and the average number of cases is one hundred a day. No less than forty physicians are reported dead, though whether they have fallen victims to the disease is not stated. The account which the correspondent gives of the disease itself, is that 'it is not cholera, but plague, with dilated pupils, carbuncles, and pestilential bubo.' Dr. Charles Murchison, physician to the London Fever Hospital, expresses, in a letter to the *Times*, his opinion that the malady is 'relapsing fever,' which, under different designations, has been well known in Britain and Ireland for nearly two centuries, which constituted a great part of the Irish epidemic of 1847, and which about the same time was very prevalent in various parts of Germany. The mortality from relapsing fever is not great, only three per cent, but Dr. Murchison shows that all epidemics of such fevers have coexisted with typhus, in which the mortality is twenty per cent. The public, he says, need be under little apprehension as to the importation of the Russian epidemic into England. The more formidable of the two diseases composing it is here already, and has, for the last three years been very prevalent among the poor of London."—*St. Petersburg Telegram to the London Times.*

BERLIN, April 7.

Authentic intelligence touching the Russian epidemic, states that three several maladies exist at the same time in St. Petersburg. In October last, *meningitis spinalis* appeared at St. Petersburg. This a spasmodic affection of the brain and spinal cord, by which children are chiefly attacked; the mortality was from twenty to fifty per cent. In November, typhus was added to the first-mentioned disease, occurring sporadically at first, and gradually developed into a malignant species of *febris recurrens.* The fever lasts a week at a time, the several attacks being separated by intervals as long. During these intervals the health is apparently so good that people have been dismissed from hospital who died soon after. A special committee has been formed, under Gov.-Gen. Suwaroff, to look after those apparently cured. On a second or third attack there is a general collapse, decomposition of blood, and paralysis. Quinine and stimulants have no effect. The deaths, at first but twenty, have risen to forty per cent. The spleen and liver are much affected. In many cases epidemical inflammation of the spleen, or *postula maligna,* has been observed. Quite recently the Siberian plague has broken out also. Of this, seventy per cent die within a few hours. A strong disposition to vomit, which cannot be satisfied, a swelling of the belly, pestilential carbuncles, and dark color of the skin, are its unmistakable symptoms. It is the "black death." St. Petersburg papers deny the existence of the plague in the capital, but the official *Northern Post* states it to have broken out at Szaniewo, in the Waldai Hills, and the description given in the St. Petersburg official *Medical News* and *Exchange News,* in which the dilation of the pupils is especially dwelt upon, shows the malady, in its present stage, greatly to resemble the plague. In many cases, indeed, it is difficult to distinguish plague from *febris recurrens,* at the time when typhoid epidemics are abroad. The disease is apparently on the decrease. Dr. Erichson, surgeon to the Emperor Nicholas, aged seventy-five, died while attending hospital. In Poland also, an epidemic has broken out. One case at Kole, near Warsaw, is represented in the *Warsaw Gazette* as *meningitis spinalis.* Out of five thousand inhabitants in that town, there are thirty-six sick and fifteen dead. In Eastern Prussia, there are many cases of *meningitis* near Dantzic.—*Correspondence of the London Times.*

---

American Medical Association.—The American Medical Association will hold its Sixteenth Annual Meeting in the City of Boston, on Tuesday, June 6th, 1865.

The following Committees are expected to report:—

On Insanity—Dr. H. R. Storer, Mass., Chairman.

On Exsection and its connection with Conservative Surgery—Dr. Lewis A. Sayres, N. Y., Chairman.

On Drainage and Sewerage of Large Cities, and their Influence on Public Health—Dr. W. J. C. Duhamel, D. C., Chairman.

On Alcohol and its Relations to Man—Dr. G. E. Morgan, Maryland, Chairman.

On Quarantine—Dr. Wilson Jewell, Penn., Chairman.

On Medical Ethics—Dr. John A. Murphy, Ohio, Chairman.

On the Microscope—Dr. James M. Corse, Penn., Chairman.

On the Relations which Electricity sustains to the Causes of Disease—Dr. Squire Littell, Penn., Chairman.

On the Morbid and Therapeutic Effects of Mental and Moral Influences—Dr. A. B. Palmer, Mich., Chairman.

On the Causes of the Extinction of the Aboriginal Races of America—Dr. Geo. V. Suckley, Md., Chairman.

On the Causes and Treatment of Ununited Fractures—Dr. Frank H. Hamilton, N. Y., Chairman.

On Diphtheria—Dr. Lucius Clark, Ill., Chairman.

On the Uses and Abuses of Pessaries—Dr. James P. White, N. Y., Chairman.

On International Medical Ethics—Dr. J. Baxter Upham, Mass., Chairman.

On Climatology and Epidemic Diseases—Dr. C. W. Parsons, R. I., Chairman.

On Autopsies in Relation to Medical Jurisprudence—Dr. T. C. Finnell, N. Y., Chairman.

On so-called Spotted Fever—Dr. James J. Levick, Penn., Chairman.

On the Introduction of Disease by Commerce, and the Means of its Prevention—Dr. A. Nelson Bell, N. Y., Chairman.

On Patent Rights and Medical Men—Dr. David Prince, Ill., Chairman.

On Revision of Plan of Organization—Dr. N. S. Davis, Ill., Chairman.

On Specialists—Dr. J. Homberger, N. Y., Chairman.

On Compulsory Vaccination—Dr. A. N. Bell, N. Y., Chairman.

On Rank of Medical Corps in the Army—Dr. C. S. Tripler, U.S.A., Chairman.

On Rank of Medical Corps in the Navy—Dr. T. L. Smith, N. Y., Chairman.

WM. B. ATKINSON, *Permanent Secretary.*

---

Œsophagism.—As an example of the nervous condition termed œsophagism, M. Nélaton, some time ago, called the attention of his class to a man of vigorous temperament, 35 years of age, and in good health, who came to the hospital under the idea that he had a foreign body in the œsophagus. A fortnight previously, while picking his teeth with a thin piece of wood, he was suddenly spoken to. His attention was turned away for an instant, and at the moment he was about to make a reply, he perceived a perfect sensation of a foreign body on the left side of the pharynx. A practitioner who was at once called in, recognized the foreign body at the spot indicated, and made some vain attempts to extract it. Extremely little pain followed, but as this afterwards increased, he came to the hospital. M. Nélaton suspected from the narrative, that no foreign body existed; and observed, that not unfrequently an unpractised finger mistakes the upper edge of the coenu of the hyoid bone for the body supposed to have been swallowed. Usually these nervous symptoms disappear at the end of three months, under suitable general treatment; but M. Nélaton referred to a case in which they manifested much greater tenacity. A lady, about six months since, being about to drink some water sweetened with syrup, not liking the appearance of the latter, placed a single drop on the tip of her tongue, and discovered it to be a solution of potass. Immediately, and notwithstanding that the drop had never been swallowed, she perceived a pain at the lateral part of the pharynx, accompanied by an impossibility of swallowing. The pain diminished, but so difficult did deglutition continue to be, that the patient required an hour to swallow a simple cup of broth, while the passage of the smallest solid body was absolutely impossible. It was believed that she was the subject of stricture of the œsophagus, until M. Nélaton passed down the largest bougies with great facility.—*Med-Chir. Review.*

---

Pennsylvania Hospital.—We learn that Dr. Francis G.

Smith has resigned the position of Physician to the Pennsylvania Hospital, which he has filled with so much credit. The Trustees have filled the vacancy thus caused, by the appointment of Dr. J. M. DaCosta, the very best selection that they could have made. On being elected to this position, Dr. DaCosta at once severed his connection with the Philadelphia Hospital, where he served very acceptably for several years.—*Med. and Surg. Reporter.*

---

Citric Acid in Diabetes.—Dr. Bélin, of l'Assomption, informs us that he has not found any remedy to act so speedily in preventing the formation of sugar, and in lessening the amount of urine, as citric acid. He mentions one case in which a marked diminution of both occurred in three or four days, and that after a few weeks scarcely a trace of sugar could be detected, whilst the quantity of urine voided became reduced to the normal standard. He does not, however, say that he has ever permanently cured a case with it.

